# Lateral Plantar Angioplasty and Reverse Sural Flap Reconstruction for Complex Heel Limb Salvage

**DOI:** 10.7759/cureus.114460

**Published:** 2026-08-13

**Authors:** Madison N Sheppard, Priyanka Mehta, Kezia Duah, Duncan Tallman, Brendan Masi, Shereece Dove, Allison K Shay, George Delis, Christian K Sabbagh, Austin L Price

**Affiliations:** 1 Medicine, Lake Erie College of Osteopathic Medicine, Bradenton, USA; 2 Medicine, Rutgers New Jersey Medical School, Newark, USA; 3 Medicine and Surgery, Ohio University Heritage College of Osteopathic Medicine, Dublin, USA; 4 Medicine, Lake Erie College of Osteopathic Medicine, Erie, USA; 5 Medicine, Touro College of Osteopathic Medicine, New York, USA; 6 Medicine, Lincoln Memorial University-DeBusk College of Osteopathic Medicine, Knoxville, USA; 7 Anatomical Sciences, St. George's University School of Medicine, St. George's, GRD; 8 Medicine, Medical College of Wisconsin, Milwaukee, USA; 9 Medicine, Nova Southeastern University, Clearwater, USA; 10 Vascular Surgery, Baptist Health Medical Center South Florida, Miami, USA

**Keywords:** diabetic foot ulcer (dfu), heel reconstruction, limb-salvage, peripheral arterial disease (pad), plantar angioplasty, revascularization, reverse sural flap, successful revascularization

## Abstract

Diabetic foot ulcers complicated by peripheral arterial disease are associated with poor wound healing and an increased risk of amputation. Heel wounds are particularly difficult to reconstruct because of their weight-bearing function and limited soft-tissue coverage. While revascularization is commonly performed for critical limb ischemia, limited evidence guides its use prior to reconstructive surgery in patients with preserved in-line arterial flow. A 58-year-old woman with insulin-dependent diabetes mellitus presented with a chronic infected plantar heel ulcer with exposed calcaneus and suspected osteomyelitis. Vascular imaging demonstrated preserved in-line flow to the foot through the dorsalis pedis artery but chronic occlusion of the distal posterior tibial and plantar arteries. Given the planned reverse sural flap reconstruction and the high perfusion demands of a weight-bearing plantar flap, a multidisciplinary team performed targeted plantar angioplasty despite adequate baseline limb perfusion. Endovascular intervention of the superficial femoral, posterior tibial, and plantar arteries improved distal plantar perfusion without complications, allowing the patient to proceed with planned soft-tissue reconstruction. This case highlights a reconstructive approach in which revascularization optimized flap perfusion rather than restored limb viability. In selected patients with complex plantar heel defects, angiosome-directed revascularization may deliver additional perfusion to support reconstructive success when flap survival is the primary goal. In carefully selected patients, targeted plantar revascularization may be considered before complex heel reconstruction, emphasizing the importance of multidisciplinary planning in limb salvage.

## Introduction

Diabetic foot ulcers (DFUs) are a common and serious complication of diabetes mellitus that are associated with substantial morbidity and an increased risk of lower extremity amputation [1-3]. The coexistence of peripheral arterial disease (PAD) further compromises tissue perfusion and impairs wound healing, increasing the risk of chronic non-healing wounds, infection, and limb loss.

Plantar heel wounds pose a particular reconstructive challenge because of the specialized weight-bearing function of the heel and its limited soft-tissue coverage [4]. Continuous weight-bearing and high mechanical stress predispose the heel to tissue breakdown, delayed healing, and reconstructive failure [4]. Compared with ulcers in other foot locations, heel ulcers are associated with prolonged healing times and poorer clinical outcomes [5]. Successful management therefore requires coordinated treatment of infection, tissue loss, vascular status, and soft-tissue reconstruction. Current treatment paradigms emphasize vascular assessment and, when indicated, revascularization before definitive reconstruction in patients with PAD and tissue loss [6,7].

Reverse sural flaps remain an important reconstructive option for complex heel defects because they provide durable coverage without the need for free tissue transfer, although successful reconstruction depends on adequate distal perfusion [8-10]. While revascularization is well established in patients with limb-threatening ischemia, its role is less clearly defined in patients with preserved in-line arterial flow who are undergoing complex reconstructive procedures. In these patients, the decision to pursue additional revascularization may instead be driven by the anticipated perfusion requirements of the planned reconstruction rather than by baseline limb viability alone.

We present the case of a patient with an infected diabetic plantar heel wound who underwent targeted plantar revascularization despite preserved in-line dorsalis pedis flow to optimize perfusion before planned reverse sural flap reconstruction. This case highlights a multidisciplinary decision-making strategy in which vascular intervention was performed to support the planned reconstruction rather than to restore baseline limb perfusion.

## Case presentation

A 58-year-old woman with insulin-dependent diabetes mellitus and a history of a chronic diabetic heel ulcer, hypertension, schizophrenia, and bipolar disorder was admitted for hypoglycemia following a mechanical fall. She additionally had a history of medication non-compliance. She had undergone incision and drainage with partial calcanectomy, followed by graft and wound vacuum-assisted closure two months prior to presentation for her chronic diabetic heel ulcer.

During admission, the patient endorsed right heel pain localized to the region of the ulcer but denied pain involving the contralateral extremity. Evaluation of her right foot revealed a right heel ulcer measuring approximately 17 x 6 x 2 cm, with exposed bone, non-viable tissue, purulent drainage, and surrounding erythema, concerning for osteomyelitis. Dorsalis pedis and posterior tibial pulses were diminished bilaterally (1+/4). No acute limb pallor or mottling was noted.

Infectious workup demonstrated polymicrobial infection. Admission blood cultures grew coagulase-negative *Staphylococcus*, viridans group *Streptococcus*, and extended-spectrum beta-lactamase (ESBL)-producing *Escherichia coli*. A superficial right foot wound culture grew ESBL *E. coli*, and a urine culture grew *Candida* species. Deep wound and tissue cultures later isolated *Enterococcus* species and *Candida albicans*. She was treated with intravenous piperacillin-tazobactam and subsequently meropenem per the infectious disease service. Subsequent surveillance blood cultures showed no growth.

Given her chronic diabetic heel ulcer and concern for inadequate perfusion to support limb salvage, vascular surgery was consulted to evaluate for revascularization. Imaging included a venous duplex negative for deep venous thrombosis bilaterally. Arterial duplex showed diffuse calcified atherosclerotic plaque bilaterally, with predominantly monophasic waveforms in the right common femoral, superficial femoral, popliteal, and tibial arteries. The initial duplex impression raised concern for severe bilateral lower extremity arterial insufficiency with possible inflow disease. Computed tomography angiography of the abdomen, pelvis, and bilateral lower extremity runoff was subsequently obtained. On independent review by vascular surgery, the iliac and superficial femoral stenoses were characterized as less than 50% and not hemodynamically flow-limiting, and the lower extremity vessels were considered patent with preserved in-line flow to the foot via the dorsalis pedis artery. The patent runoff was not considered the primary driver of poor wound healing.

Limb salvage and surgical planning

The patient's foot had preserved in-line arterial flow via the dorsalis pedis artery, and on conventional criteria, additional pedal revascularization would not have been required for baseline limb viability. However, given the extent of plantar tissue loss and a collaborative reconstructive plan with plastic surgery for a reverse sural flap with external fixation, a decision was made to pursue more aggressive revascularization than baseline perfusion alone would warrant. The plastic surgery team specifically requested dedicated plantar arterial angiography to assess plantar-level patency and to maximize pedal perfusion prior to flap coverage, with the goal of supporting flap viability over a weight-bearing surface.

Plantar and lower extremity angioplasty

**Figure 1:**
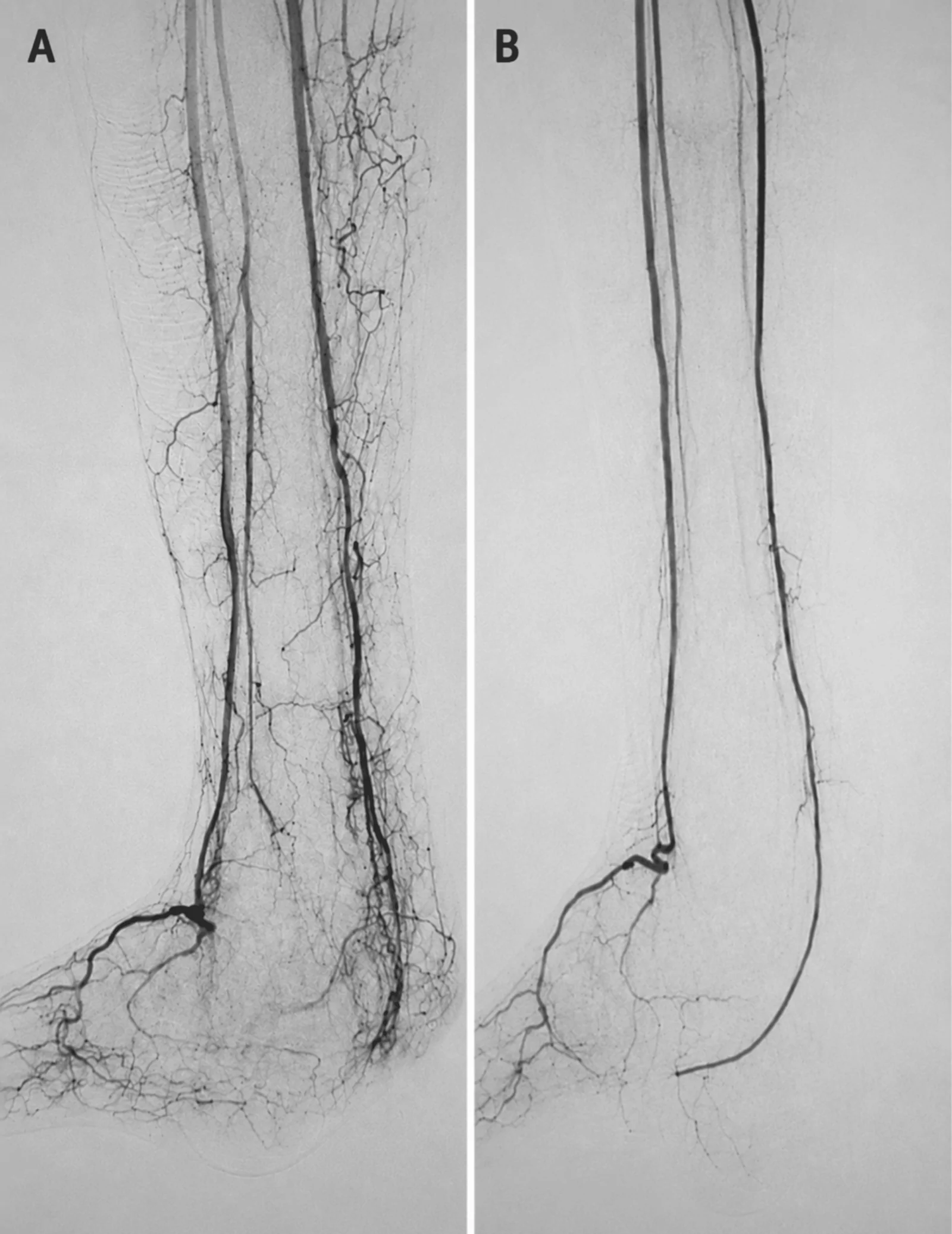
Digital subtraction angiography of the right lower extremity before and after endovascular intervention. (A) Pre-intervention angiogram demonstrating chronic total occlusion of the distal posterior tibial artery below the ankle, with diminished distal plantar filling. (B) Post-intervention angiogram after angioplasty of the posterior tibial and plantar arteries, demonstrating restored in-line flow into the plantar arch with improved distal plantar filling.

On hospital day 11, the patient underwent diagnostic angiography with intervention. Access was obtained via ultrasound-guided micropuncture of the left common femoral artery, with a 5-French sheath upsized to a 6-French 45 cm sheath. A contralateral up-and-over approach was used across the aortic bifurcation, and the patient was systemically heparinized. Angiography demonstrated a patent aortoiliac segment without hemodynamically significant stenosis. On the right, the superficial femoral artery showed greater than 70% stenosis. The posterior tibial artery was patent proximally and in the mid-segment, with a 100% chronic total occlusion below the ankle extending into the plantar arteries, characterized as a complex lesion (Figure 1A).

The right superficial femoral artery was treated with a 4 x 100 mm drug-coated balloon. The right posterior tibial and plantar occlusion was crossed using a 0.014-inch wire with a CXI support catheter (Cook Medical, Bloomington, IN, USA), confirmed intraluminal, and treated with angioplasty using a 2 x 40 mm balloon. Repeat angiography demonstrated a successful response with improved distal plantar filling (Figure 1B). The access site was closed using a 6-French Angio-Seal vascular closure device (Terumo Interventional Systems, Somerset, NJ, USA), achieving excellent hemostasis. Estimated blood loss was 10 mL, with no complications. At the conclusion of the procedure, the patient had a palpable superficial femoral pulse distal to the access site and dorsalis pedis and posterior tibial Doppler signals bilaterally.

The patient remained hemodynamically stable throughout the postoperative period without any immediate complications. The following day, she was cleared for surgical reconstruction from a vascular standpoint and was continued on dual antiplatelet therapy and a statin. She was subsequently transferred to the plastic surgery service for the planned reverse sural flap reconstruction with external fixation.

## Discussion

The central management question in this case was whether pedal revascularization should be performed despite preserved in-line flow to the foot. Conventional indications would argue against intervention: the limb was not ischemic, the angiographic disease was not believed to be the primary cause of impaired healing, and revascularization carries procedural risk. Reconstruction of a large plantar heel defect, however, introduced a different physiologic problem: whether baseline perfusion was sufficient to support flap survival. The relevant question was not whether the limb could survive, but whether the planned reconstruction could reliably heal.

That distinction reframes the indication. Contemporary limb salvage practice emphasizes restoring perfusion before definitive reconstruction in patients with PAD and tissue loss, typically by reestablishing at least one direct in-line vessel to the foot [6,7]. That principle was developed for ischemic, threatened limbs, where the objective of revascularization is limb viability itself. This patient did not fit that paradigm. The objective here was reconstruction, and a flap over a weight-bearing heel in an infected diabetic field is among the more perfusion-sensitive reconstructions in the foot. The reverse sural artery flap provides durable single-stage coverage without microsurgical free tissue transfer and is frequently chosen in comorbid patients with limited reconstructive alternatives [10]. Its perfusion is retrograde, dependent on peroneal artery perforators and the overall adequacy of distal lower extremity circulation, and flaps in this location and in diabetic, vasculopathic patients carry meaningfully elevated rates of venous congestion and partial necrosis [10]. Successful reconstruction therefore depends not on global limb perfusion but on adequate perfusion within the vascular territory responsible for flap support and wound healing.

The vascular anatomy explains why baseline in-line flow was not considered sufficient. The plantar heel is supplied principally through the posterior tibial and plantar arterial systems rather than the dorsalis pedis, so preserved dorsalis pedis flow, while adequate for baseline limb survival, does not necessarily provide sufficient perfusion to the plantar territory in which this wound and its intended flap coverage lie [4]. Treating the below-ankle posterior tibial and plantar occlusion was thus a territory-directed decision, drawing on the angiosome concept, in which revascularization is directed at the specific arterial territory supplying a wound. We do not cite the angiosome literature as evidence that intervention improves outcomes in non-ischemic limbs; that evidence derives almost entirely from patients with critical limb ischemia, and direct angiosome-targeted revascularization remains debated even within that population, with some series emphasizing technical success or collateral adequacy over strict territory targeting [11]. Rather, we invoke it as the best available physiologic framework for matching perfusion to reconstructive demand in a setting where direct evidence is lacking.

That margin is where the risk-benefit balance must be weighed explicitly. Infrapopliteal and pedal intervention is not without hazard. The diabetic pedal circulation is characterized by small vessel caliber and diffuse calcification, and intervention carries recognized risks of arterial perforation, distal embolization, and access vessel injury, with reported pedal access complication rates of approximately 5% even in experienced hands [12]. In a limb that is not acutely threatened, these risks are accepted despite the absence of an immediate limb threat in exchange for a potential perfusion advantage that can benefit the patient only if it improves the conditions for flap survival and wound healing. The procedural risk of a technically successful, ultrasound-guided intervention was judged acceptable because flap failure over an infected, weight-bearing heel risked prolonged wound care, additional operations, or limb loss.

The corollary matters as much as the argument. Absent a planned reconstruction of this demanding kind, pedal revascularization in a limb with preserved in-line flow would be difficult to justify, and that course should not be inferred from this report. The decision was reasonable here precisely because of the conjunction of factors: extensive plantar tissue loss, active infection, and a perfusion-dependent flap over a weight-bearing surface, managed across vascular surgery, plastic surgery, orthopedic stabilization, wound care, and infectious disease [6]. The threshold for revascularization may differ when the clinical objective is reconstruction rather than limb salvage. In selected patients with advanced plantar tissue loss and a planned, perfusion-sensitive flap, revascularization beyond the threshold required for baseline limb viability may be justifiable to support reconstruction, provided the procedural risk is low and the target territory is the one on which the reconstruction depends.

This report has limitations. It describes the vascular evaluation and the revascularization decision through the point of perfusion optimization and handoff to the reconstructive team. The reverse sural flap, the external fixation, and the longer-term outcomes of flap viability, wound healing, and ambulation were beyond the scope of the documented course presented here. Prospective reporting of these downstream outcomes-flap survival, time to healing, and return to ambulation on the reconstructed heel-would help establish whether the perfusion advantage sought here translates into measurable reconstructive benefit, and we regard such data as an important direction for future study. This report therefore supports a decision-making framework rather than demonstrating the efficacy of the intervention, and the perfusion strategy it describes should be read as a defensible plan grounded in physiologic reasoning and the available literature, not as evidence of healing benefit in this patient.

## Conclusions

This case illustrates a revascularization decision made to support reconstruction rather than to restore limb viability. In a patient with preserved in-line flow to the foot through the dorsalis pedis artery, targeted plantar revascularization was undertaken to optimize perfusion within the angiosome on which a planned reverse sural flap would depend. Accordingly, in carefully selected patients with extensive plantar tissue loss and a planned, perfusion-sensitive flap, the threshold for revascularization may reasonably differ from that applied to limb salvage alone. This case documents the reasoning behind that decision rather than its outcome.
